# Deep sequencing and automated histochemistry of human tissue slice cultures improve their usability as preclinical model for cancer research

**DOI:** 10.1038/s41598-019-56509-5

**Published:** 2019-12-27

**Authors:** Susann Haehnel, Kristin Reiche, Dennis Loeffler, Andreas Horn, Conny Blumert, Sven-Holger Puppel, Nicole Kaiser, Felicitas Rapp, Michael Rade, Friedemann Horn, Juergen Meixensberger, Ingo Bechmann, Frank Gaunitz, Karsten Winter

**Affiliations:** 1 Institute of Anatomy, University of Leipzig, Faculty of Medicine, Leipzig, Germany; 2 Department of Diagnostics, Fraunhofer Institute of Cell Therapy and Immunology, Leipzig, Germany; 3 GSI Helmholtzzentrum für Schwerionenforschung GmbH, Darmstadt, Germany; 4 Institute of Clinical Immunology, University of Leipzig, Faculty of Medicine, Leipzig, Germany; 5 Department of Neurosurgery, University Hospital Leipzig, Leipzig, Germany

**Keywords:** CNS cancer, Cancer models

## Abstract

Cancer research requires models closely resembling the tumor in the patient. Human tissue cultures can overcome interspecies limitations of animal models or the loss of tissue architecture in *in vitro* models. However, analysis of tissue slices is often limited to histology. Here, we demonstrate that slices are also suitable for whole transcriptome sequencing and present a method for automated histochemistry of whole slices. Tumor and peritumoral tissue from a patient with glioblastoma was processed to slice cultures, which were treated with standard therapy including temozolomide and X-irradiation. Then, RNA sequencing and automated histochemistry were performed. RNA sequencing was successfully accomplished with a sequencing depth of 243 to 368 x 10^6^ reads per sample. Comparing tumor and peritumoral tissue, we identified 1888 genes significantly downregulated and 2382 genes upregulated in tumor. Treatment significantly downregulated 2017 genes, whereas 1399 genes were upregulated. Pathway analysis revealed changes in the expression profile of treated glioblastoma tissue pointing towards downregulated proliferation. This was confirmed by automated analysis of whole tissue slices stained for Ki67. In conclusion, we demonstrate that RNA sequencing of tissue slices is possible and that histochemical analysis of whole tissue slices can be automated which increases the usability of this preclinical model.

## Introduction

Cancer constitutes an enormous burden on societies worldwide. Despite achievements, rendering some types of cancer curable, the overall occurrence of cancer is increasing because of growth and aging of populations^1^. Research on cancer, aiming at the development of new drugs and therapeutic strategies requires models that most closely resemble the *in vivo* situation in a patient in order to have a predictive value for future treatment. Today, most models are based on (immortalized) cell lines grafted into immunosuppressed animals. Their relevance is further hampered by interspecies limitations between humans and rodents. During the last years, organotypic slice cultures derived from human tissues, including tumors, came into focus as an alternative model^2^. These models may become a valuable alternative to animal testing not only reducing the numbers of experimental animals but also overcoming interspecies differences. In our group, we have already established slice cultures from human brains^3^, *Glioblastoma multiforme* (GBM)^4,5^, head and neck squamous cell carcinoma^6^, human gastric and esophagogastric junction cancer^7^, and colorectal carcinoma^8^. Using these organotypic slice cultures, we tested, for example, effects of heavy ion therapy^5^, polyethylenimine-based nanoparticles for siRNA delivery^9^, but also novel nanostructured scaffolds for cultivation^4^.

A prerequisite to use such models as clinical test system for the outcome of therapy or the selection of the most effective drug for individual patients is an unbiased, fast and automated cell counting approach allowing to start treatment within a couple of days. Moreover, whole transcriptome analysis with and without treatment would be of help for prediction, but also to better understand mechanisms of tumor progression and therapy resistance.

In order to address these two important issues, we focused on GBM slice cultures which maintain their histopathological hallmarks for at least 14 days *in vitro*^5^. GBM is the most common primary brain malignancy in adults^10^ with a median survival of approximately 15 months^11,12^ despite surgical resection, X-irradiation and chemotherapy with temozolomide (TMZ). We report that organotypic slice cultures are suitable for automated histological analyses as well as whole transcriptome sequencing, thereby providing an adequate alternative with regard to individualized cancer research and therapy.

## Results

### Tissue integrity is maintained in slice cultures during 13 days of cultivation

**Figure 1:**
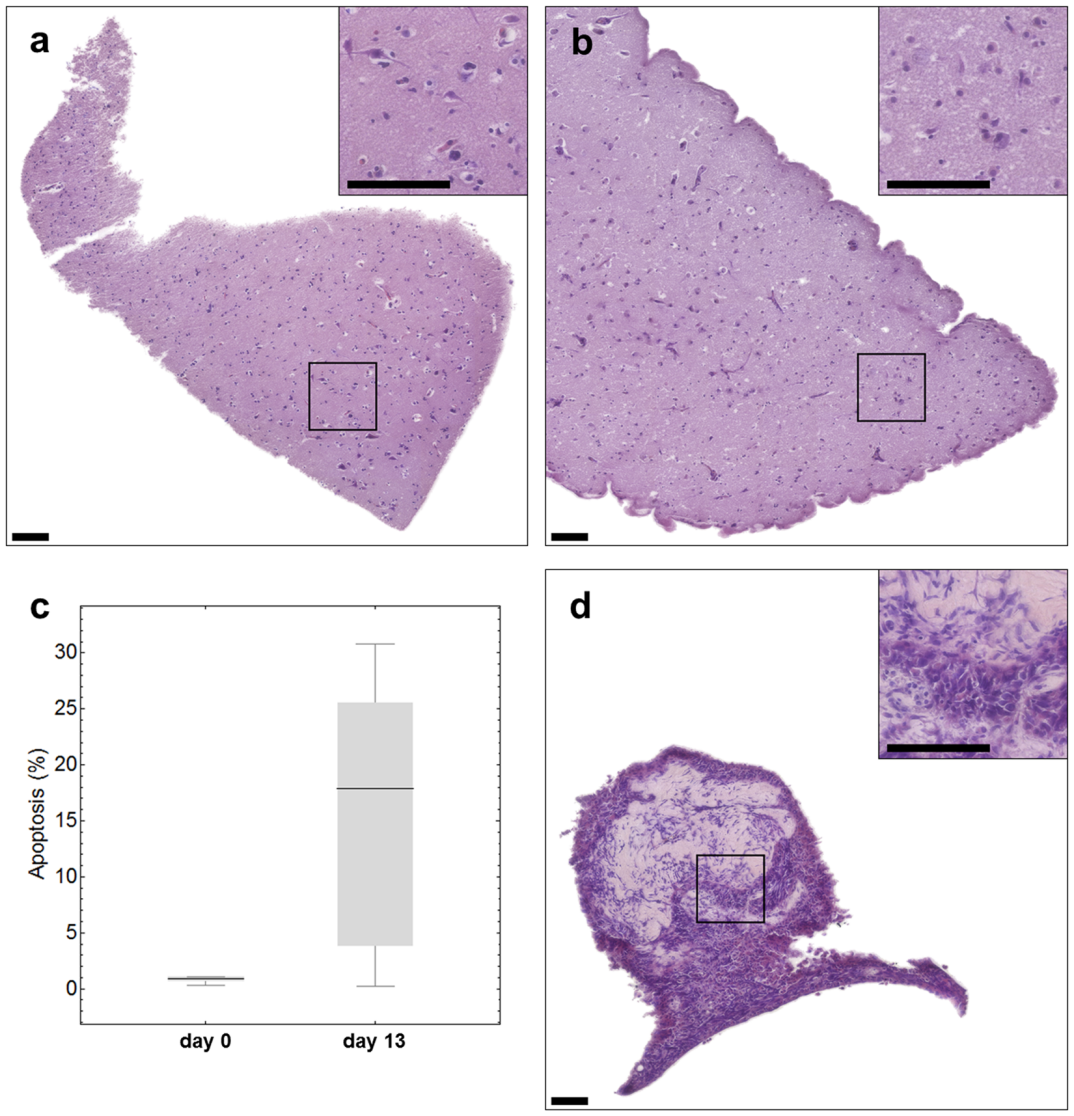
Histology of freshly sliced (**a**) or cultivated (**b**) tumor-surrounding brain tissue (peritumoral tissue of zone III) and cultivated GBM tissue (**d**). Hematoxylin (nuclei) and eosin (cytoplasm) staining was done (**a**) instantly after the slicing procedure or after 13 days in culture (**b,d**). Apoptosis rate was determined by TUNEL staining in peritumoral tissue on day 0 (left bar) and day 13 (right bar) (**c**). Scale bar: 100 µm.

In order to see whether cultivation had an influence on tissue integrity, hematoxylin and eosin staining of tissue slices was performed immediately after preparation and after cultivation for 13 days. As can be seen in Fig. 1, the cell density of freshly cut peritumoral brain tissue of zone III (Fig. 1a) decreases after 13 days of cultivation (Fig. 1b). In addition, we observed an increase of apoptotic cells from 1% on day 0 to 17% on day 13 (Fig. 1c, p = 0.034). Despite an obvious loss of cells, this result also indicates that the tissue is maintained to a high degree. In Fig. 1d tumor tissue after 13 days of cultivation is presented. Unfortunately, the amount of material obtained from the patient was very limited. Therefore, we were not able to present a comparison of the tumor tissue from day 13 to day 0. But, it should be noted that we have previously demonstrated that the individual histopathology of tissue cultures derived from glioblastoma is maintained over at least 16 days^5^.

### RNA obtained from tissue slices is suitable for whole transcriptome sequencing

**Figure 2:**
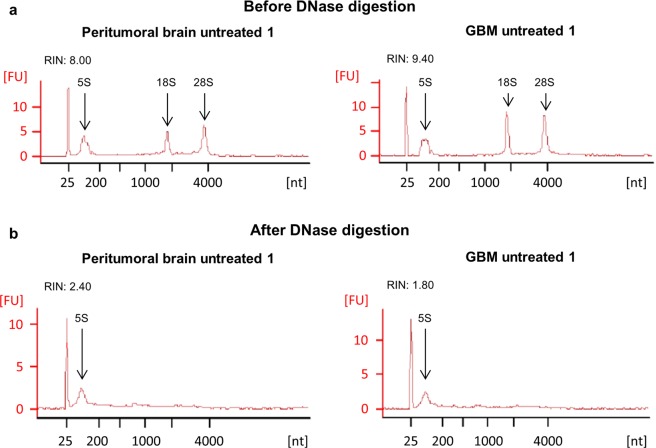
RNA quality of cultivated tissue slices. RNA quality was determined by a Bioanalyzer 2100 using the RNA 6000 Nano-Kit (Agilent Technologies) and revealed good quality before the DNase digestion was performed (**a**). After the DNase digestion, the RNA quality was strongly reduced (**b**). The left graphs show untreated peritumoral brain tissue, the right graphs the corresponding GBM tissue.

Next, we asked whether the RNA isolated from treated and untreated tissue slices can be further used for whole transcriptome sequencing. Therefore, RNA was isolated from peritumoral brain (zone III) and GBM tissue (zone I) either treated with TMZ and X-irradiation or left untreated. For each condition, the RNA isolated from three individual slices pooled together was collected in order to have enough material for further analyses and to overcome the tumor’s heterogeneity. Using a Bioanalyzer 2100, the RNA integrity number (RIN) was determined from each sample before and after DNase digestion. The corresponding data are presented in Table 1. The higher the RIN value, the better is the RNA maintenance^13^. As can be seen in Table 1, all RIN values were ≥ 7 before the DNase digestion which demonstrates a very high RNA quality (Table 1). After DNase digestion, a severe loss of RNA quality was observed as indicated by strongly diminished RIN values (the reason for that is not known, but a contamination of the utilized chemicals with RNase could be excluded in further analyses). This loss of quality was further indicated by a loss of the characteristic peaks of the 18 s and 28 s rRNA in the corresponding chromatograms (Fig. 2). Only the peak of the 5 s rRNA still was clearly distinct (Fig. 2b). Although RNA quality seemed to be insufficient for whole transcriptome sequencing as concluded from the RIN values determined, it should be noted that higher RINs are only necessary for transcriptome sequencing of poly(A) RNA. In our experiments total RNA sequencing was performed which even allows using RNA from FFPE tissue with RINs worse than those presented in our data^14–16^. In fact, next generation sequencing was performed successfully. Library preparation and sequencing resulted in sequencing depths from 243 to 368 x 10^6^ reads per sample. For unknown reasons, this was not the case for one duplicate of treated peritumoral brain tissue (zone III) although respective RIN values were even better than those obtained from other slices (Table 1). Our data clearly demonstrate that whole transcriptome sequencing from slice cultures is possible.

**Table 1:** RNA integrity number before (left) and after (right) DNase digestion.

Sample	RNA Integrity Numbers	
	before DNase digestion	after DNase digestion
Tumor_untreated_1	9.40	1.80
Tumor_untreated_2	8.60	1.20
Tumor_TMZ+4Gy_1	9.20	2.40
Tumor_TMZ+4Gy_2	9.20	2.40
Peritumoral brain_untreated_1	8.00	2.10
Peritumoral brain_untreated_2	7.80	1.20
Peritumoral brain_TMZ+4Gy_1	8.40	2.40
Peritumoral brain_TMZ+4Gy_2	9.10	2.50

### Technical replicates reveal a high consistency of sequencing data

**Figure 3:**
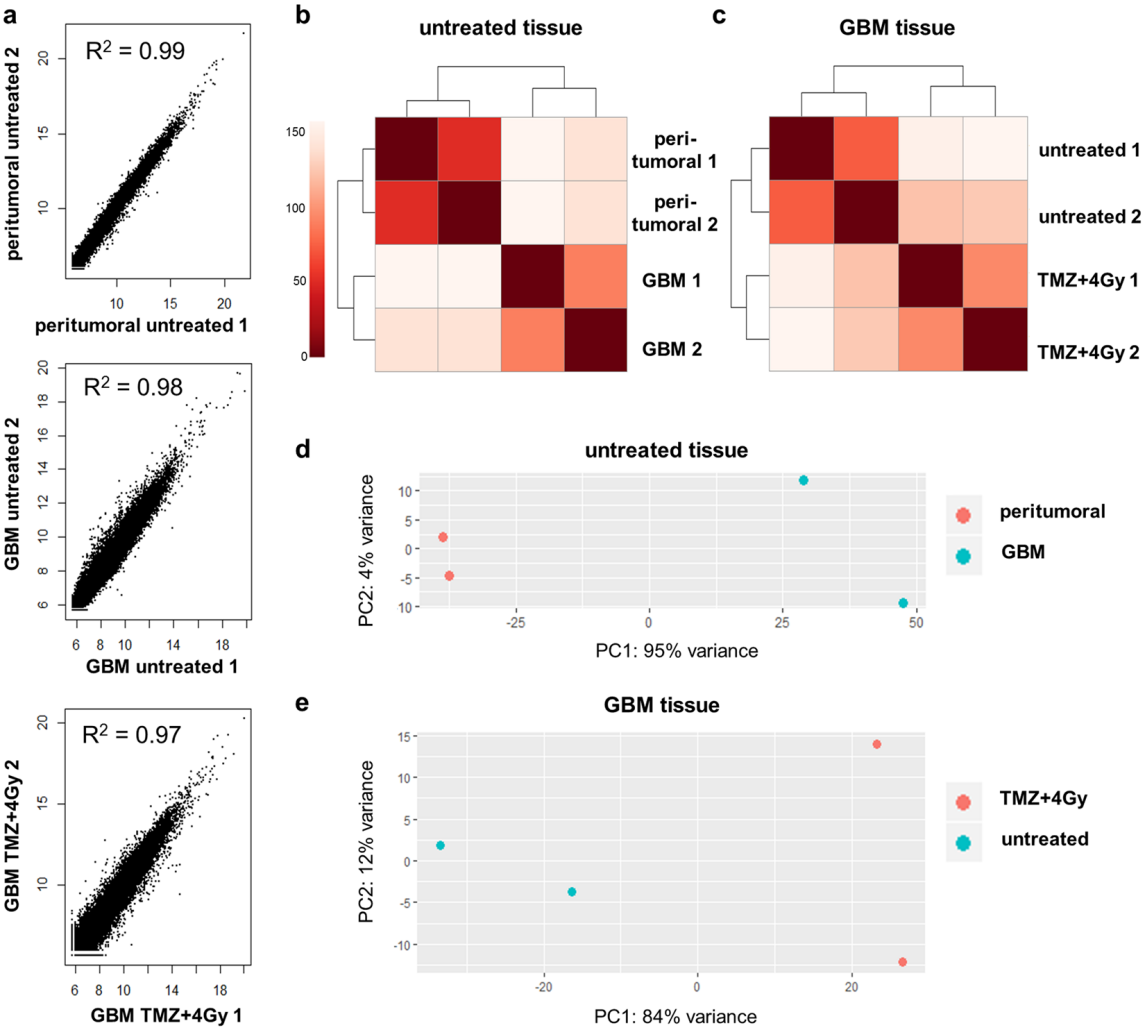
Comparison of gene expression between peritumoral brain and GBM tissue. (**a**) Correlation plots of variance-stabilized counts in sample duplicates (peritumoral untreated = untreated peritumoral brain tissue of zone III, GBM untreated = untreated GBM tissue of zone I, GBM TMZ + 4 Gy = GBM tissue treated with radiochemotherapy). The correlation coefficient represents low variability between duplicates. (**b**) Distance heatmap of Euclidean distances between untreated peritumoral brain (peritumoral) and GBM tissue (GBM). (**c**) Distance heatmap of Euclidean distances between untreated and treated (TMZ + 4 Gy) GBM tissue. (**d**) Principal Component Analysis. Untreated sample duplicates cluster together with a high variability between peritumoral brain and GBM tissue. (**e**) Principal Component Analysis. GBM sample duplicates show differences between untreated and treated (TMZ + 4 Gy) GBM tissue.

As described in the preceding paragraph, the whole transcriptome sequencing from RNA isolated from tissue slices was successful. The next question to be answered was how consistent the results were among individual experimental replicates. To this end, the data obtained by separate sequencing experiments from two slice pools (three slices were pooled in each approach) for each condition and tissue type were compared. The linear correlation coefficient R^2^ of variance-stabilized counts was calculated for each pair (Fig. 3a). For all three sample pairs, the correlation coefficient was close to 1, so that a linear correlation between the duplicates could be assumed. As expected, the variance within the GBM samples was slightly higher than in the peritumoral brain samples (Fig. 3a,d) probably due to high intra-tumor heterogeneity which is well-known for GBM^17^. The heatmaps (R package “pheatmap” with default parameters) of the pairwise Euclidean distances of variance-stabilized counts show that the sample duplicates cluster together but clearly separate from the other tissue samples and conditions (Fig. 3b,c). The principal component analysis of the variance-stabilized counts confirmed these findings (Fig. 3d,e). The variance between peritumoral brain and GBM tissue was higher (Fig. 3d) than between treated and untreated GBM tissue (Fig. 3e).

### Differential gene expression between peritumoral brain (zone III) and GBM tissue (zone I) and between treated and untreated GBM tissue

By the experiments presented in the preceding paragraphs it could be confirmed that the data obtained by whole transcriptome sequencing are reliable, since expression variation was reproducible between duplicates of two different tissue cultures of the same patient. To gain further insight into differential gene expression between peritumoral brain (zone III) and GBM tissue (zone I) and between treated and untreated GBM tissue, a differential gene expression analysis was done.

**Figure 4:**
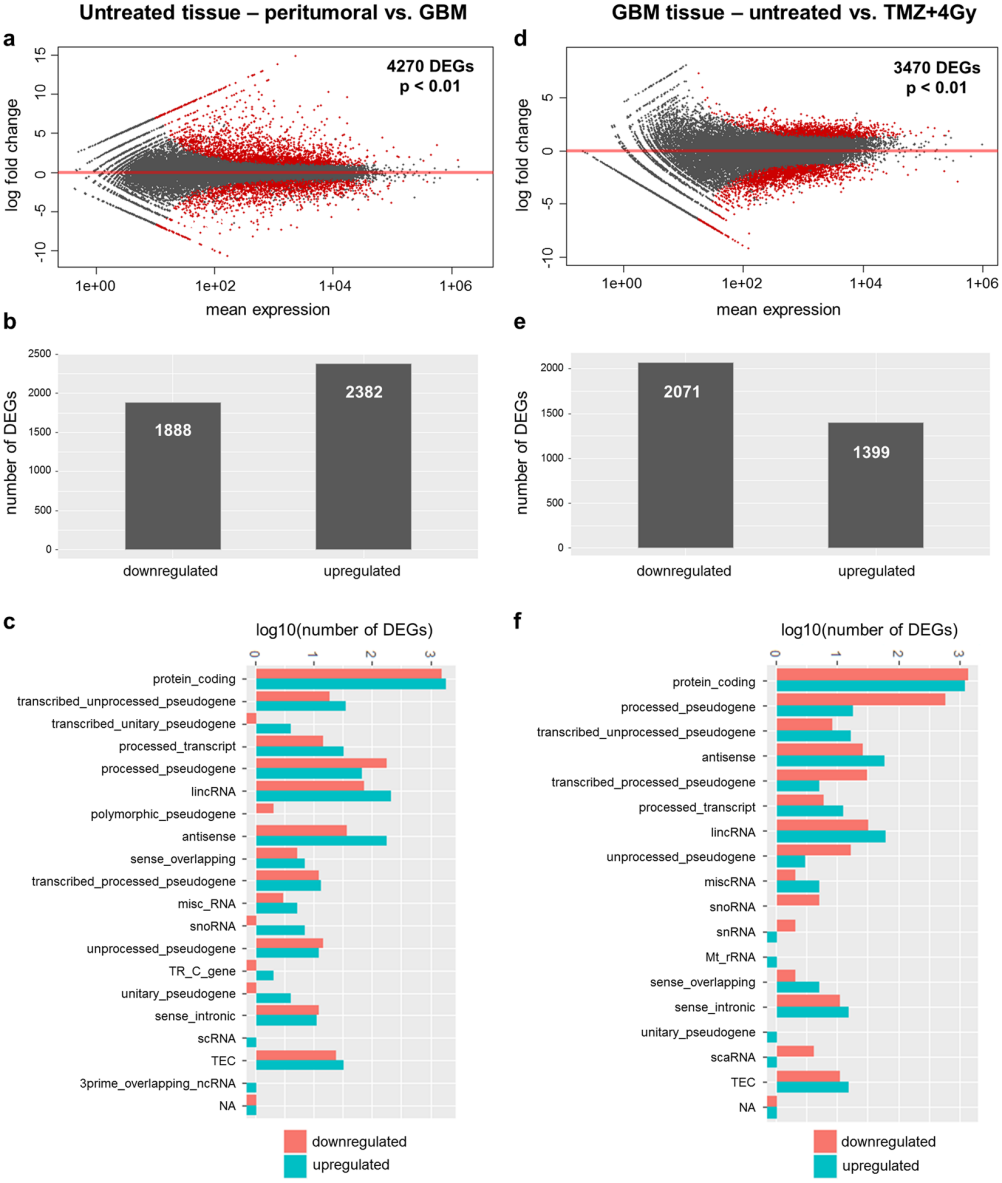
Differentially expressed genes (DEGs) between peritumoral brain and GBM tissue. Analysis of differentially expressed genes (DEGs) between untreated peritumoral brain tissue of zone III and GBM tissue samples of zone I (**a–c**) and between untreated and treated GBM samples (**d–f**). (**a,d**) Significantly regulated transcripts are indicated in red (p < 0.01). (**b,e**) Number of down- and upregulated genes in both comparisons. (**c,f**) Biotype of down- (red) and upregulated (blue) transcripts in both comparisons. TEC = to be experimentally confirmed, NA = not available.

A calculation with DESeq2 revealed 4270 significantly differentially (FDR < 0.01) regulated transcripts between untreated peritumoral brain (zone III) and GBM tissue (zone I, Fig. 4a). 1888 of these DEGs were found to be significantly downregulated, and 2382 genes were significantly upregulated in the tumor tissue (zone I) in comparison to the peritumoral brain (zone III, Fig. 4b). The vast majority of all DEGs belonged to the protein-coding fraction of transcripts (Fig. 4c). In addition, known human pseudogenes and non-coding RNAs represented approximately 100 DEGs both in the downregulated and in the upregulated transcripts. A corresponding comparison of untreated versus treated GBM tissue (Fig. 4f) revealed 3470 significantly regulated (FDR < 0.01) transcripts. Here, 2071 DEGs were found to be significantly downregulated and 1399 significantly upregulated in GBM tissue which had been treated in contrast to untreated samples (Fig. 4d,e).

A pathway enrichment analysis by the Ingenuity® Pathway Analysis software tool (Qiagen) revealed that the vast majority of the protein-coding genes which are significant differentially expressed between untreated peritumoral brain and GBM tissue and between untreated and treated GBM tissue are known to be associated with certain diseases and/or biological functions. Tables 2 and 3 show an excerpt of these diseases and functions with the corresponding p-values and the numbers of molecules present in both datasets of differentially expressed protein-coding genes. In peritumoral brain versus GBM tissue, 3040 of the 3280 differentially expressed protein-coding transcripts were found to be associated with the tumorigenesis of tissue (Table 2). 511 transcripts are known to play a role in cellular growth and proliferation (Table 2). In untreated versus treated GBM tissue, 2189 of the 2527 protein-coding transcripts are associated with tumorigenesis of tissue and 778 were found to be associated with cellular function and maintenance (Table 3). Further significantly enriched functions are, among others, cell death, cell and organismal survival, proliferation of tumor cells, progression of cell cycle, and cell-to-cell signaling (Tables 2 and 3).

**Table 2:** Top diseases and functions of significant differentially expressed genes in untreated peritumoral brain vs. GBM tissue.

Diseases and disorders	p-value	molecules of 3280 in total
Cancer	1.64 × 10^−09^ − 1.29 × 10^−150^	3101
- Tumorigenesis of tissue	3.29 × 10^−145^	3040
- Malignant solid tumor	1.90 × 10^−139^	3084
Organismal injury and abnormalities	1.64 × 10^−09^ − 1.29 × 10^−150^	3135
Gastrointestinal disease	8.04 × 10^−10^ − 1.88 × 10^−130^	2822
Endocrine disorders	1.47 × 10^−09^ − 3.03 × 10^−112^	2641
Dermatological diseases and conditions	6.23 × 10^−11^ − 3.08 × 10^−90^	1926
**Molecular and cellular functions**		
Cellular development	9.97 × 10^−10^ − 1.91 × 10^−44^	598
Cellular growth and proliferation	9.97 × 10^−10^ − 1.91 × 10^−44^	511
- Proliferation of neuronal cells	3.25 × 10^−15^	198
Cellular assembly and organization	4.76 × 10^−10^ − 1.90 × 10^−41^	747
Cellular function and maintenance	9.97 × 10^−10^ − 1.90 × 10^−41^	973
Cell-to-cell signaling and interaction	1.65 × 10^−09^ − 1.60 × 10^−37^	677
**Physiological system development and function**		
Nervous system development and function	1.65 × 10^−09^ − 1.91 × 10^−44^	994
Tissue development	1.65 × 10^−09^ − 1.91 × 10^−44^	999
Embryonic development	1.61 × 10^−09^ − 8.66 × 10^−40^	768
Organismal development	1.65 × 10^−09^ − 8.66 × 10^−40^	1198
Tissue morphology	1.61 × 10^−09^ − 7.98 × 10^−33^	804

**Table 3:** Top diseases and functions of significant differentially expressed genes in treated vs. untreated GBM tissue.

Diseases and disorders	p-value	molecules of 2527 in total
Cancer	6.38 × 10^−04^ − 1.00 × 10^−63^	2306
- Tumorigenesis of tissue	1.29 × 10^−62^	2189
- Malignant solid tumor	4.32 × 10^−58^	2263
- Glioma	5.59 × 10^−04^	216
Organismal injury and abnormalities	6.38 × 10^−04^ − 1.00 × 10^−63^	2327
Gastrointestinal disease	4.31 × 10^−04^ − 2.00 × 10^−56^	2148
Hepatic system disease	4.31 × 10^−04^ − 2.09 × 10^−39^	1632
Reproductive system disease	1.93 × 10^−04^ − 2.98 × 10^−36^	1514
**Molecular and cellular functions**		
Gene expression	5.07 × 10^−08^ − 6.05 × 10^−13^	514
Cellular assembly and maintenance	6.38 × 10^−04^ − 1.13 × 10^−12^	525
Cellular function and maintenance	3.19 × 10^−04^ − 1.13 × 10^−12^	406
Cell death and survival	6.22 × 10^−04^ − 1.36 × 10^−10^	778
Cell cycle	5.47 × 10^−04^ − 5.51 × 10^−10^	348
- Cell cycle progression	5.51 × 10^−10^	251
- Proliferation of tumor cells	6.38 × 10^−04^	99
**Physiological system development**		
Organismal survival	9.88 × 10^−13^ − 1.30 × 10^−13^	554
Nervous system development and function	5.53 × 10^−04^ − 9.90 × 10^−12^	380
Tissue morphology	5.53 × 10^−04^ − 1.18 × 10^−09^	268
Organ morphology	4.31 × 10^−04^ − 1.10 × 10^−08^	255
Organismal development	4.35 × 10^−04^ − 1.10 × 10^−08^	551

### Knowledge base analysis of expression data predicts reduced proliferation in slices after treatment which could be confirmed by automated histochemical analysis

**Figure 5:**
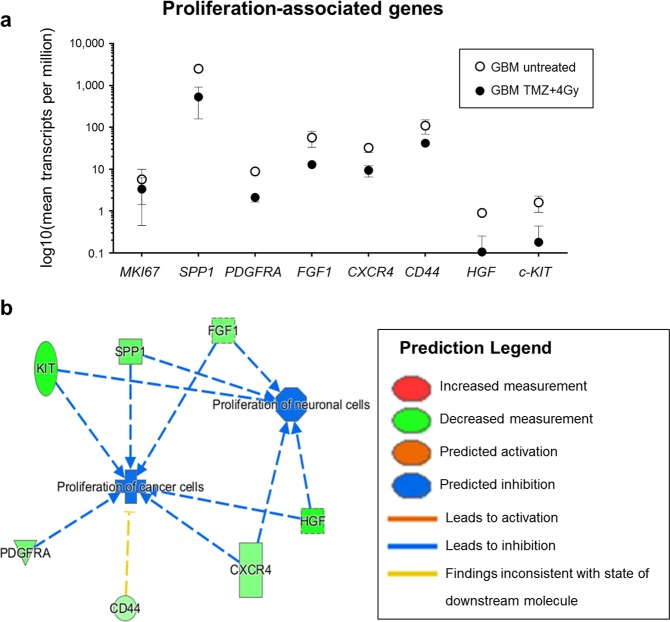
mRNA expression indicates an inhibition of proliferation after treatment. The differentially expressed transcripts in treated versus untreated GBM tissue were compared to a list of proliferation-associated genes obtained from the Ingenuity® Pathway Analysis (IPA®, QIAGEN). 190 genes were found to be present in both lists. Transcripts per million of some of these genes are displayed in (**a**). Knowledge base analysis with IPA® indicates an inhibition of proliferation of neuronal and cancer cells (**b**, blue lines). Green symbols represent a decreased measurement of the respective transcript.

In the previous sections it was demonstrated that whole transcriptome sequencing can be performed with tissue slices in order to reveal differences in gene expression. Now it was of interest, whether these data can be used to make predictions about possible physiological responses to treatment that can be confirmed by a second method. Therefore, we performed a knowledge base data analysis using the Ingenuity® Pathway Analysis (IPA®) software tool (Qiagen). An IPA®-generated list of genes which are described to be associated with proliferation of cancer and/or neuronal cells was compared to the significantly regulated transcripts that were found between treated and untreated GBM tissue. The analysis revealed 190 genes that were present in both lists. Further analysis indicated reduced proliferation under treatment conditions (Fig. 5b). Among the most prominent genes we identified down-regulation of *MKI67*, *SPP1*, *PDGFRA*, *FGF1*, *CXCR4*, *CD44*, *HGF* and *KIT* under the influence of treatment (Fig. 5a).

**Figure 6:**
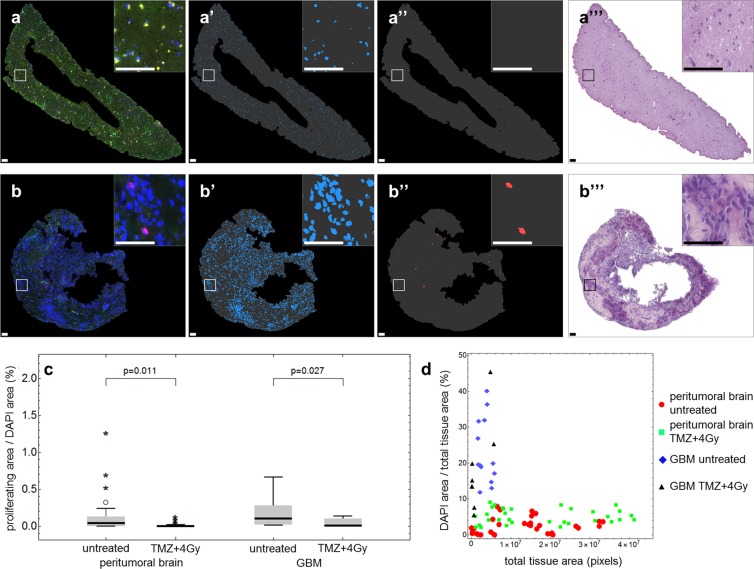
Histological finding of reduced proliferation after treatment supports mRNA expression data. Paraffin-embedded treated and untreated peritumoral brain (**a**) and GBM tissue (**b**) was stained with a Ki67 antibody as proliferation marker (red) and DAPI as nuclei marker (blue) and recorded by a slide scanner. Representative images of untreated samples are presented. (Note: green signals are attributed to autofluorescence of the tissue). For quantification, the total tissue area (a’/”, b’/”, gray), the nuclei area (a’, b’) and the Ki67-positive nuclei area (a”, b”) were determined. H/E stainings of consecutive tissue sections are shown in a”’ and b”’. (**c**) Ratio of proliferating area (Ki67- and DAPI-positive pixel area) per DAPI area in untreated and treated (TMZ + 4 Gy) peritumoral brain (left) and GBM tissue (right). (**d**) Ratio of DAPI area per total tissue area compared to total tissue area in pixels in untreated and treated (TMZ + 4 Gy) peritumoral brain (red circles, green squares) and GBM tissue (blue diamonds, black triangles). Biological replicates: 1; Technical replicates: 3; Scanned sections: 33 (untreated peritumoral brain), 32 (treated peritumoral brain), 13 (untreated GBM), 8 (treated GBM). Scale bars: 100 µm (a), 50 µm (**b**).

In order to confirm a negative effect on proliferation in the tumor slices of this patient under treatment, as predicted by gene expression analysis, we performed immunohistochemistry on paraffin sections derived from slices. For the analysis, a quantitative image analysis was implemented. In the experiment presented in Fig. 6, slices from peritumoral brain (zone III, Fig. 6a) and from GBM tissue (zone I, Fig. 6b) were labeled with an antibody directed against Ki67 (untreated samples are shown as example). Ki67 is a commonly used proliferation marker which is present during G1, S, G2, and mitosis but absent in G0 phase^18^. In addition, DAPI was used to counterstain nuclei in order to evaluate whether a Ki67-positive signal is indeed localized to a nucleus to prevent counting of unspecific signals. Figures 6a,b show the original pictures recorded by the slide scanner. In a first step, the pixel area of the whole tissue was calculated (gray masks in Fig. 6a’/’’,b’/’’) as well as the DAPI-positive area (Fig. 6a’,b’) representing the nuclei. To determine the proliferation capacity of peritumoral brain (Fig. 6a) and GBM tissue (Fig. 6b), double-positive nuclei were analyzed (Fig. 6a’’,b’’). Consecutive H/E-stained sections of the tissue are shown in Fig. 6a’’’,b’’’ to demonstrate the native condition of the analyzed tissue slices. The automatic quantification revealed a statistically significant decrease of proliferating cells in treated peritumoral brain and GBM tissue compared to the untreated controls (Fig. 6c). Furthermore, GBM tissue has a high nuclei density and a small tissue area, whereas peritumoral brain tissue exhibits a larger tissue area combined with a smaller cellular density (Fig. 6d).

The results of the automated analysis were confirmed by manual analysis. Segmented areas of total tissue and DAPI were highly correlated (R^2^ = 0.998 and R^2^ = 0.876, respectively; all p < 0.001) while values for the proliferating area showed moderate correlation (R^2^ = 0.616, p < 0.001) (Fig. S3).

## Discussion

Despite intense research during the last decades, many cancerous diseases are still associated with a poor prognosis and a low median overall survival, e.g. 14 months for advanced non-small cell lung cancer^19^, 12 months for advanced gastric cancer^20^, and 15 months for GBM^12^. Therefore, the establishment of preclinical models to test newly developed drugs and treatment strategies is an important step in oncological research. As outlined in the introduction, the frequently used animal models often fail because of interspecies differences that impede clinical translation. Cell culture models, on the other hand, are far away from the *in vivo* situation as tumor tissue can be composed of a bulk of many other cell types aside from tumor cells, e.g. endothelial cells^21^, pericytes^22^, tumor-associated immune cells^23^, and cancer stem-like cells^24^ which is not reflected by cell culture models. As a more realistic system patient-derived xenograft models have been developed, injecting patient-derived tumor cells into immunodeficient mice^25^. Thus, the animals generate tumors which are supposed to maintain the original tumor’s biology thereby mimicking the human patient. This is, among others, well described for breast cancer^26^, non-small cell lung cancer^27^, or melanoma metastasis^28^. Besides the great burden for the animals, the production of patient-derived tumors within rodents is a time-consuming method which is therefore unlikely to find its way into a clinical setting with regard to personalized cancer therapy. The immunodeficiency of these mice, which is required to inhibit the rejection of injected human tumor cells^29,30^ further impedes the successful translation into the clinics.

As an alternative to animal and cell culture models, human tissue slice cultures are now increasingly employed in cancer research^2,31–37^. One of the major advantages of tissue slice cultures is the maintenance of the tissue topology and composition of different cell types including immune cells, as represented by microglia which play a crucial role in GBM progression^38–40^. Therefore, slice cultures may reflect the tumor’s heterogeneity far better than conventional cell culture and animal models. Yet, tumor heterogeneity is not only defined by the general presence of different cell types, but also by different characteristics of the tumor cells in different areas of the tissue^41^. This impedes the reproducibility of such *ex vivo* experiments and increases the difficulty of successful translation into a clinical setting for human patients. For that reason, the slices obtained from one patient are pooled together and are randomly distributed in triplicates to the membrane inserts. For RNA analysis, these slices are pooled again to diminish the possibility that the differences observed here are just resulting from a different localization within the original tumor.

For histology, single slices are embedded in paraffin and stained individually. In conventional microscopy, only parts of the whole tissue can be recorded and analyzed. Furthermore, most histological analyses are still performed “manually” which is time-consuming and investigator-dependent. In this study, as exemplified by tissue from one GBM patient, we present that whole slices can be recorded and analyzed automatically (Fig. 6). Therefore, it is possible to retrospectively draw conclusions about the extent of heterogeneity in the original tissue. The automation of the histological analysis is time-saving, objective and reproducible. That in turn increases the suitability for a clinical application of this method with regard to individualized cancer therapy. By designing the experiments in duplicate or even triplicate approaches (depending on the available amount of tissue) the results are getting even more reproducible. In addition, the RNA expression analyses presented here were performed in replicates and exhibited a very good correlation and only slight differences within each sample pair (Fig. 3). Therefore, it can be concluded that the random distribution of three slices may be sufficient to depict the intratumoral heterogeneity. Further investigations on more GBM slice cultures are currently being analyzed to confirm this finding and to verify whether this is consistent among patients.

The histological finding of reduced proliferation in treated GBM tissue is consistent with RNA expression data obtained from the same samples. Here, the same treatment-mediated effect was observed (Fig. 5a). Eight genes, which were found to be downregulated in treated compared to untreated GBM tissue and are known to be associated with proliferation of neuronal and/or cancer cells, were chosen for further analysis. This analysis revealed a downregulation of *SPP1* which has been shown to be overexpressed in grade IV gliomas and which is related to worse overall survival also in patients with lower-grade glioma^42^. Some isoforms of *SPP1* are in fact known to promote glioma cell invasion^43^. In addition, we identified a down-regulation of *CD44* under treatment (Fig. 5c). This down-regulation may be caused by down-regulation of *SPP1* which was shown to increase the synthesis of the CD44 variant *CD44v6* in liver cancer cells^44^. CD44 itself is known as a marker of GBM invasiveness and was shown to promote stem cell-like properties in glioma and to play a role in the mediation of resistance to radiation and chemotherapy with temozolomide^45,46^. An increased expression of *CXCR4* is associated with the recurrence of glioblastoma after radiochemotherapy and could indicate an activation of the CXCL12-CXCR4 pathway representing an alteration in the angiogenic pattern within the tumor^47^. *FGF1* and other members of the FGF family are involved in cell proliferation, differentiation, and migration^48^. Therefore, down-regulation of these family members is in agreement with the histologically observed decrease of proliferation. At this point, it is also interesting to note that FGF1/FGFR signaling activates Aurora A, a kinase which is involved in the maintenance of the stem cell characteristics of GBM cells^49^. We further found down-regulation of *PDGFRA* and *c-KIT* which is especially interesting as these receptor tyrosine kinases have long been suggested as GBM therapeutic targets^50,51^. In conclusion, the treatment-induced changes in mRNA expression are in agreement with the histological analysis which demonstrated inhibition of proliferation, as determined by a statistically significant decrease in the Ki67-positive pixel area under treatment (Fig. 6c).

The confirmation of the automatic analysis procedure was done by manual segmentation by three independent observers and both approaches were correlated with each other. A certain divergence of values among the three observers was noticed. While the results for total tissue area were very consistent, there was a notable spread in results for DAPI area which could be attributed to blooming around the stained nuclei. These minimal blooming artifacts appear during image acquisition and have no impact on the automatic analysis. Nevertheless, they proved to be interfering for observers during manual analysis. The large spread for the proliferating area was mainly caused by low signal intensities, poor image contrast and faintly remaining background fluorescence. These factors generally impede manual analysis and observers tend to underestimate threshold values. Overall, there was a very good correlation between manually and automatically obtained results for the total tissue area, which could be easily segmented by the three observers. The comparison of manual and automatic analysis of the DAPI area also showed very good correlation, although a manual under-segmentation was noted. The corresponding comparison of the proliferation area determination exhibited a moderate correlation and results indicated a manual over-segmentation. Values from individual images showed notable dispersion between automatic and manual analysis.

In conclusion, our data, in compliance with former studies^4–7^ demonstrate that organotypic slice cultures provide a suitable model for mimicking the *in vivo* situation within the patient thereby allowing insights into tumor biology that would not be possible by the use of conventional cell culture or animal models. By this means, it helps to reduce the numbers of animals used in cancer research. Furthermore, it may promote the way to individualized cancer medicine which is the current goal for therapeutic approaches. In the future and with the simultaneous development of new drugs it could be conceivable to prepare slice cultures for each patient, test possible chemotherapeutics and assist the physicians concerning the individual treatment strategy^2,36,52^.

## Material and Methods

### Patient and samples

Glioblastoma tissue was obtained by surgery of a 51 year old male patient diagnosed with primary glioblastoma (GBM, WHO grade IV). Surgery and diagnosis were performed at the Department of Neurosurgery and the Department of Neuropathology, University Hospital Leipzig, Germany, according to the EANO guideline for the diagnosis and treatment of anaplastic gliomas and glioblastoma^53^. To get surgical access to the MRI contrast-enhanced tumor tissue ( = zone I), also tumor-surrounding brain tissue had to be removed. In the following, we refer to the tumor-surrounding tissue as peritumoral tissue ( = zone III), which is basically normal brain tissue with only very few tumor cells^54^. Both tissue types were subjected to organotypic tissue slice cultures in duplicates. Tissue acquisition and experimental procedure were approved by the institutional research ethics board (Ethical Review Committee of the Medical Faculty of the University of Leipzig, #144-2008; registration numbers: IORG0001320, IRB00001750) in accordance with the Helsinki Declaration (https://www.wma.net/policies-post/wma-declaration-of-helsinki-ethical-principles-for-medical-research-involving-human-subjects/). The patient provided written informed consent for experimental usage of his tissue samples and retrospective analysis of the data according to the General Data Protection Regulation of the European Community (https://gdpr-info.eu/).

### Tissue slice preparation

**Figure 7:**
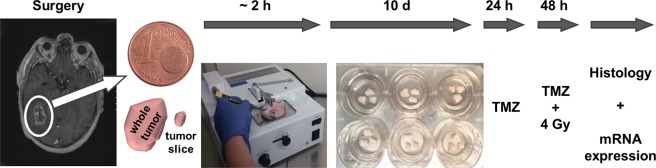
Experimental setup. Freshly resected glioblastoma (zone I) and peritumoral brain tissue (zone III) was transported into the lab in sterile transport medium and stored at 4 °C. The production of 350 µm tissue slices was performed with a tissue chopper. The slices were separated from each other by the wide opening of a glass pipette and randomly allocated to membrane inserts and put in the wells of sterile 6-well plates, previously filled with 1 ml of cultivation medium. The slices were cultivated 10 days before treatment with radiochemotherapy was implemented. 24 hours prior to irradiation with 4 Gy the slices were pretreated with 200 µM temozolomide (TMZ). After a total treatment time of 72 hours the slices were either fixed in 4% paraformaldehyde for histological analyses or processed for RNA and protein isolation to perform whole transcriptome sequencing and protein analyses. We acknowledge Dr. Sonja Kallendrusch (Institute of Anatomy, University of Leipzig, Faculty of Medicine, Germany) who kindly provided the photograph of the tissue chopper.

Tissue slices that can be maintained in culture for at least 14 days were prepared using a previously described protocol^5^. In brief, surgically removed tissue not required for neuropathological diagnostic was transferred to Dulbecco’s Modified Eagle Medium (DMEM, Gibco) supplemented with glucose (4.5 g/l, Gibco), fetal calf serum (10%, Biochrom), Glutamax (1%, Gibco) and penicillin/streptomycin (1%, Gibco). Organotypic tissue slices were prepared using a tissue chopper (McIlwain TC752) under sterile conditions (Fig. 7). Before preparation, a razor blade was sterilized by autoclaving. A normal glass pipette as well as a glass pipette with the fine tip broken off and appropriate forceps were autoclaved. The tissue was washed twice with fresh Minimum Essential Medium (MEM, Gibco) and was put on a stack of sterile filter membranes, cut into ~ 350 µm thick slices and transferred into ice-cold MEM. The slices were separated from each other by pipetting up and down with the wide opening of the broken-off glass pipette. Using this pipette they were randomly transferred onto membrane culture inserts (Millipore) in triplicates. The inserts were put into six-well plates equipped with 1 ml medium per well. The culture medium was composed of MEM, 25% Hank’s Balanced Salt Solution (with Ca^2+^ and Mg^2+^, ThermoFisher Scientific), 10% heat-inactivated horse serum (Gibco), 1% L-glutamine (Gibco), 1% glucose (Mediatech Inc.) and 1% penicillin/streptomycin (Gibco). The slices were cultivated on a liquid/air interface in a humidified incubator at 37 °C and 5% CO_2_ for 13 days in total. During cultivation, slices were provided with fresh medium every 2 to 3 days.

### Treatment of tissue slices

After 10 days in culture, slices were treated with temozolomide (TMZ, 200 µM). Control slices were incubated with the corresponding amount of dimethyl sulfoxide (DMSO, 0.2% v/v) used as vehicle. 24 hours after initial treatment, slices were X-irradiated (4 Gy) or sham-irradiated (control slices), and provided with fresh TMZ- or DMSO-supplemented medium the other day. For X-irradiation, a 200 kV irradiation machine (Gulmay Medical D3000, Gulmay, Surrey, UK) with a copper filter was used. The dose rate was 1.156 Gy/minute and each sample was irradiated 3.46 minutes to reach the target dose of 4 Gy. After a total treatment time of 72 hours, slices were processed for further analyses (Fig. 7).

### Histology

Slices were fixed in 4% paraformaldehyde at 4 °C overnight and washed with phosphate-buffered saline (PBS). Slices were dehydrated and embedded in paraffin. Paraffin sections (7 µm) were cut with a sledge microtome and collected on glass slides (3 sections per slide). Hematoxylin and eosin staining was performed to evaluate the tissue maintenance. Photographs were taken with a digital slide scanner (Pannoramic Scan II, 3D HISTECH Ltd., Budapest, Hungary).

For immunological staining, every third slide per condition was dewaxed in xylene and rehydrated in decreasing concentrations of ethanol. Before immunostaining, the slides were pretreated two times for 20 minutes with citrate buffer (pH 6) in a microwave. Slides were washed with PBS and permeabilized/blocked with 0.3% Triton/PBS and 10% normal goat serum for 30 minutes. The primary antibody against Ki67 (MIB1 clone, mouse, 1:100, Dako, code number: M7240) was diluted in 0.3% Triton/PBS with 1% normal goat serum and incubated overnight at 4 °C. The Alexa 568-labeled secondary antibody (goat anti-mouse, 1:800, Gibco, catalog number: A-11004) was diluted in PBS and slides were incubated for 1 hour at room temperature. To stain the nuclei, slides were incubated with DAPI (ThermoFisher Scientific) for 15 minutes at room temperature. Slides were thoroughly washed with PBS and aqua dest. and covered with Fluorescence Mounting Medium (Dako) and coverslips. For apoptosis detection, five to six slides per condition were dewaxed as described above. A TUNEL assay was performed according to the manufacturer’s protocol (Click-iT™ Plus TUNEL Assay, Alexa Fluor™ 594, Invitrogen™, order number C10618). To stain the nuclei, slides were incubated with DAPI, washed, and covered with coverslips as described above.

### Imaging and image analysis

The immunofluorescently stained microscope slides were fully digitized at 20x magnification using a digital slide scanner (Pannoramic Scan II, 3D HISTECH Ltd., Budapest, Hungary) equipped with a quad band (DAPI/FITC/TRITC/Cy5) filter set. DAPI filter was used for blue DAPI channel, FITC filter was used for green tissue autofluorescence channel, and TRITC filter was used for Ki67 channel. Images of the stained tissue slices were exported from slide scanner data sets (Pannoramic Viewer, Version 1.15.4, 3D HISTECH Ldt., Budapest, Hungary) as PNG images with pixel dimensions of 0.325 µm. Some regions in the exported images had to be masked by hand (Adobe Photoshop CS6, Adobe Systems Inc., San Jose, USA) in order to remove artifacts (i.e. tissue overlaps, air bubbles, unspecific staining, dirt/fluorescent particles, blooming, etc.). Spectral bleedthrough between different color channels was corrected using the “Spectral Unmixing” plugin for ImageJ (Version 1.51n, http://imagej.hih.gov/ij). Image analysis was performed with Mathematica (Version 11.1, Wolfram Research, Inc., Champaign, IL, USA). Corrected fluorescence images were imported and split into separate color channels. In order to obtain tissue masks (almost entirely represented by DAPI and autofluorescence signals), all images were smoothed with a 5 pixel wide Gaussian filter and binarized using Otsu’s (cluster variance maximization) thresholding method^55^ prior to color channel separation. DAPI signals within blue image channels were also binarized using Otsu’s thresholding method while proliferation marker (Ki67) signals within red image channels were binarized using Kapur’s (histogram entropy minimization) thresholding method^56^. Since specific proliferation marker staining can only occur within the nuclei, the binarized DAPI and Ki67 images were multiplied in order to omit unspecific staining outside of nuclei. The resulting masks were further cleared of very small segments (up to 20 pixels) to eliminate specks of fluorescent particles within nuclei. Finally, the areas of total tissue, DAPI and Ki67 masks were determined and ratios were computed. Numbers of analyzed images were as follows: 33 for untreated peritumoral brain tissue, 32 for peritumoral brain treated with TMZ + 4 Gy, 13 for untreated GBM tissue, 8 for GBM tissue treated with TMZ + 4 Gy.

To verify the result of the automated image analysis approach we performed an additional interactive analysis by three independent observers using ImageJ. Corrected fluorescence images were imported and split into separate color channels (DAPI, Ki67, autofluorescence). Subsequently, all color channels were segmented by interactive thresholding. Manually generated masks were imported in Mathematica and analyzed corresponding to the automatically segmented masks. Calculated parameters of the three observers’ segmentations were averaged and ratios were computed.

Tissue slices with apoptosis staining underwent the same imaging and image preprocessing procedures as the microscope slides stained against Ki67, as mentioned above. Apoptosis was captured using the TRITC filter of the digital slide scanner. Spectral unmixing was performed and apoptosis signals within red image channels were bianrized using Kapur’s (histogram entropy minimization) thresholding method. Binarized DAPI and apoptosis images were multiplied in order to omit unspecific staining outside of nuclei. Subsequently, segmented images were inspected and masked by hand if necessary (e.g. vessels, artifacts). Finally, the areas of total tissue, DAPI, and apoptosis masks were determined, ratios were computed, and results were averaged for all slices originating from the same tissue slice.

### RNA sequencing

Total RNA from cultivated tissue slices was isolated using the miRNeasy mini Kit (Qiagen) following the provided manufacturer’s protocol. RNA yield was measured with the Qubit 2.0 instrument (Life Technologies) using the RNA Broad Range Assay. Total RNA amount per sample ranged from 1.5 to 2.9 µg. RNA quality was determined by the Bioanalyzer 2100 using the RNA 6000 Nano-Kit (Agilent Technologies). All samples had RNA integrity numbers of ≥ 7.6 (Table 1, before DNase digestion). RNA was DNase-digested twice using the TURBO DNA free Kit (Ambion®, ThermoFisher Scientific).

For library preparation with the Truseq-Stranded Total RNA Sample Prep Kit (Illumina) up to 200 ng RNA per sample were used. A ribosomal RNA (rRNA) depletion step using the Ribo-Zero Gold rRNA Removal Kit (Illumina) was conducted according to the manufacturer’s protocol and – depending on the quality of each sample – a fragmentation was done. Every library was equipped with two barcodes to allow multiplexing of the samples. Concentrations were determined using the Qubit DNA Kit and the DNA quality was detected by the Bioanalyzer 2100 (DNA1000 Kit). According to the average size, which is determined by the Bioanalyzer, and the exact concentration of the samples, the molarity of each library was calculated.

The samples were sequenced at the HiSeq2500 with 2 × 126 bp paired-end reads. 12 pM of DNA were put on the flowcell using one lane per sample. The number of reads obtained was between 243 and 368 × 10^6^ reads per sample, except for one sample (“peritumoral brain TMZ + 4 Gy 2”) with less than 50,000 reads.

### Data analysis and statistics

#### Primary and secondary data analysis

Postprocessing of obtained raw reads per sample included demultiplexing using Illumina bcl2fastq v1.84 and secondary data analysis covering adaptor trimming, read mapping and expression quantification. Data processing of the secondary data analysis was invoked and monitored by the universal analysis pipeline (http://uap.readthedocs.io/en/master/), ensuring consistent and reproducible execution of each single analysis step per sample. The according configuration files are available as Supplementary File S1. In detail, adaptor sequences (adaptor 1: AGATCGGAAGAGCACACGTCT, adaptor 2: AGATCGGAAGAGCGTCGTGTA) were removed from raw reads by utilizing AdaptorRemoval v.2.2.0^57^ with additional parameters –trimns –trimqualities –minquality 20, and –minlength 20 in order to trim terminating ambiguous bases or bases with a quality score less than 20 and to discard reads shorter than 20 bases. Trimmed reads were mapped to the human reference genome version GRCh38/hg38 by segemehl v0.2.0^58^ in split read mode (option –splits) and with additional parameters –hitstrategy 1 and –differences 1 to report the best alignment with at maximum one indel or mutation in the initial seed and passing the default minimal alignment accuracy. Expression quantification for the human reference gene annotation Gencode v25^59^ was obtained by using HTSeq v0.6.1^60^ with parameters –stranded = reverse, –type = exon, –idattr = gene_id and –mode = intersection-strict. The number of reads assigned to a gene is, thus, defined by the number of paired reads that completely map to the exons of this gene and that do not map to any other gene. For assessing expression variation among samples raw counts were variance-stabilized by using the R library DeSeq2 version 1.10.1^61^. For visualization of expression, data raw gene counts were transformed to transcripts per million (TPMs) in order to correct for different sequencing depths of RNA libraries and gene length.

#### Quality control of obtained deep sequencing data

In order to assess the overall quality of the RNA sequencing for each tissue specimen a subsample of 1 million raw paired-end reads was randomly chosen by fastq-sample v0.0.14 (http://hannonlab.cshl.edu/fastx_toolkit/) using default parameters (https://github.com/dcjones/fastq-tools). Each sample was evaluated according to the following criteria using FastQC v0.11.5 (https://www.bioinformatics.babraham.ac.uk/projects/fastqc/), FastQ Screen v0.11.1a (https://www.bioinformatics.babraham.ac.uk/projects/fastq_screen/), and self-developed scripts (for details see Supplemental Methods): (i) minimal Illumina Phred Quality Score of 30 reflecting minimal base call accuracy of 99.9%, (ii) no adapter sequence remnants detected, (iii) a negligible number of reads mapped to reference genomes other than human, and (iv) more than 90% of reads mapped to the human reference genome GRCh38/hg38 (Fig. S1). A manually assorted list of human rRNA sequences (see S1 Table for NCBI RefSeq identifiers) was used to calculate the fraction of reads mapping to human rRNA transcripts, resulting in fractions ranging from 17% to 66% (Fig. S2).

All samples except one (“peritumoral brain TMZ + 4 Gy 2”) passed all quality criteria (Figs. S1 and S2). For the remaining samples, a high fraction of reads mapping to rRNA transcripts was observed. However, reads corresponding to endogenous rRNA resulted in a maintainable number of reads. The fraction of high reads mapping antisense to rRNA genes resembled rRNA antisense probes from the rRNA depletion step, and thus do not affect assessment of transcriptome variation (Fig. S2).

#### Differential expression analysis

Differential expression was assessed with negative binomial models by using the R library DESeq2 version 1.10.1^61^ and RStudio version 1.1.442^62^. Both Samples of the treated peritumoral brain tissue (“peritumoral brain TMZ + 4 Gy”) were excluded from differential expression analysis because minimal number of required sample size was not reached due to sequencing failure of one sample of this group. The linear term for the negative binomial model to obtain significant changes in gene expression between two selected contrasts of interest (untreated peritumoral brain vs. untreated GBM tissue, untreated GBM vs. treated GBM tissue) is:

$$log{\lambda }_{gi}={\beta }_{0}+{\beta }_{1}\cdot grou{p}_{k}$$

with *λ*_*gi*_ denoting the relative abundance of gene *g* in sample *i*. The group parameter *group*_*k*_ reflects a vector specifying the contrasts used for expression variation assessment. It assigns samples to the groups “untreated peritumoral brain” and “untreated GBM tissue” or to the groups “treated GBM” and “untreated GBM”, respectively. For both contrasts, expression variation was assessed for all genes with at least one read count in all regarded samples. Default settings of independent filtering of the DeSeq2 R library were used. All genes with a false discovery rate (FDR) < 0.01^63^ were classified to be significantly differentially expressed.

#### Ingenuity® pathway analysis (IPA®)

The pathway enrichment analysis was done with the Ingenuity® Pathway Analysis software tool version 44961306 (IPA®, Qiagen). A table containing all the significant differentially expressed transcripts of the protein-coding fraction between treated and untreated GBM samples (2527 transcripts) and between untreated GBM versus peritumoral brain samples (3280 transcripts) was uploaded. A core analysis was run with default parameters based on expression log ratio. To link the histological data to the expression analysis data, a list of genes which are well-known to be associated with the proliferation of cancer and/or neuronal cells, was generated by IPA®. This IPA® list (1678 genes) was compared to the list of significant differentially expressed protein-coding genes between treated and untreated GBM tissue and the number of transcripts present in both lists was calculated. Of the 190 genes which were found in both lists, 7 of the most prominent ones were chosen for further analyses. They were extracted from the list of differentially expressed genes (DEGs) between treated and untreated GBM tissue, another core analysis was run with default parameters and the z-score was calculated. The z-score indicates whether an associated disease, function or pathway is predicted to be inhibited or activated under the given expression values^64^. Figure 5 shows the results of this analysis. Green gene symbols in the figure illustrate the measured downregulation of the gene and blue arrows indicate the inhibition of the corresponding biological function, representing negative z-scores calculated by IPA®.

### Statistical analysis of image quantification data

Statistical analysis was performed with IBM SPSS Statistics (version 22; IBM Corp.; Armonk, New York, USA). Data were tested for normal distribution using the Shapiro-Wilk test. Group comparisons were performed using Kruskal-Wallis test with Dunn’s post hoc tests to adjust the p-value for multiple comparisons. Correlation analysis of manually and automatically calculated values was performed by computing Spearman’s rank correlation coefficient. Significance for all tests was set at p < 0.05. Data were expressed as median and interquartile range, boxplots and scatterplots were generated using Mathematica.

## Supplementary information

Supplementary Figures

Supplementary Tables

Supplementary Methods

Supplementary Information

## Data Availability

The deep sequencing datasets generated and analyzed during the current study are available in the GEO repository GSE119102. The histological datasets generated during the study are available from the corresponding author on reasonable request.
