# Supplementary material for: Deep sequencing and automated histochemistry of human tissue slice cultures improve their usability as preclinical model for cancer research
Source: Sci Rep. 2019 Dec 27;9:19961. doi: 10.1038/s41598-019-56509-5 (PMC6934722; doi:10.1038/s41598-019-56509-5)
Supplement: Supplementary file 1 — Supplementary Figures [file 41598_2019_56509_MOESM1_ESM.pdf]

Deep sequencing and automated histochemistry of human tissue slice cultures improve their usability as preclinical model for cancer research

Susann Haehnel[1*], Kristin Reiche[2], Dennis Loeffler[2], Andreas Horn[1], Conny Blumert[2], Sven-Holger Puppel[2], Nicole Kaiser[1], Felicitas Rapp[3], Michael Rade[2], Friedemann Horn[4,2], Juergen Meixensberger[5], Ingo Bechmann[1], Frank Gaunitz[5+] and Karsten Winter[1+]

[1]Institute of Anatomy, University of Leipzig, Faculty of Medicine, Germany

[2]Department of Diagnostics, Fraunhofer Institute of Cell Therapy and Immunology, Leipzig, Germany

[3]GSI Helmholtzzentrum für Schwerionenforschung GmbH, Darmstadt, Germany

[4]Institute of Clinical Immunology, University of Leipzig, Faculty of Medicine, Germany

[5]Department of Neurosurgery, University Hospital Leipzig, Germany

*corresponding author: su.haehnel@gmail.com

[+]contributed equally to this work

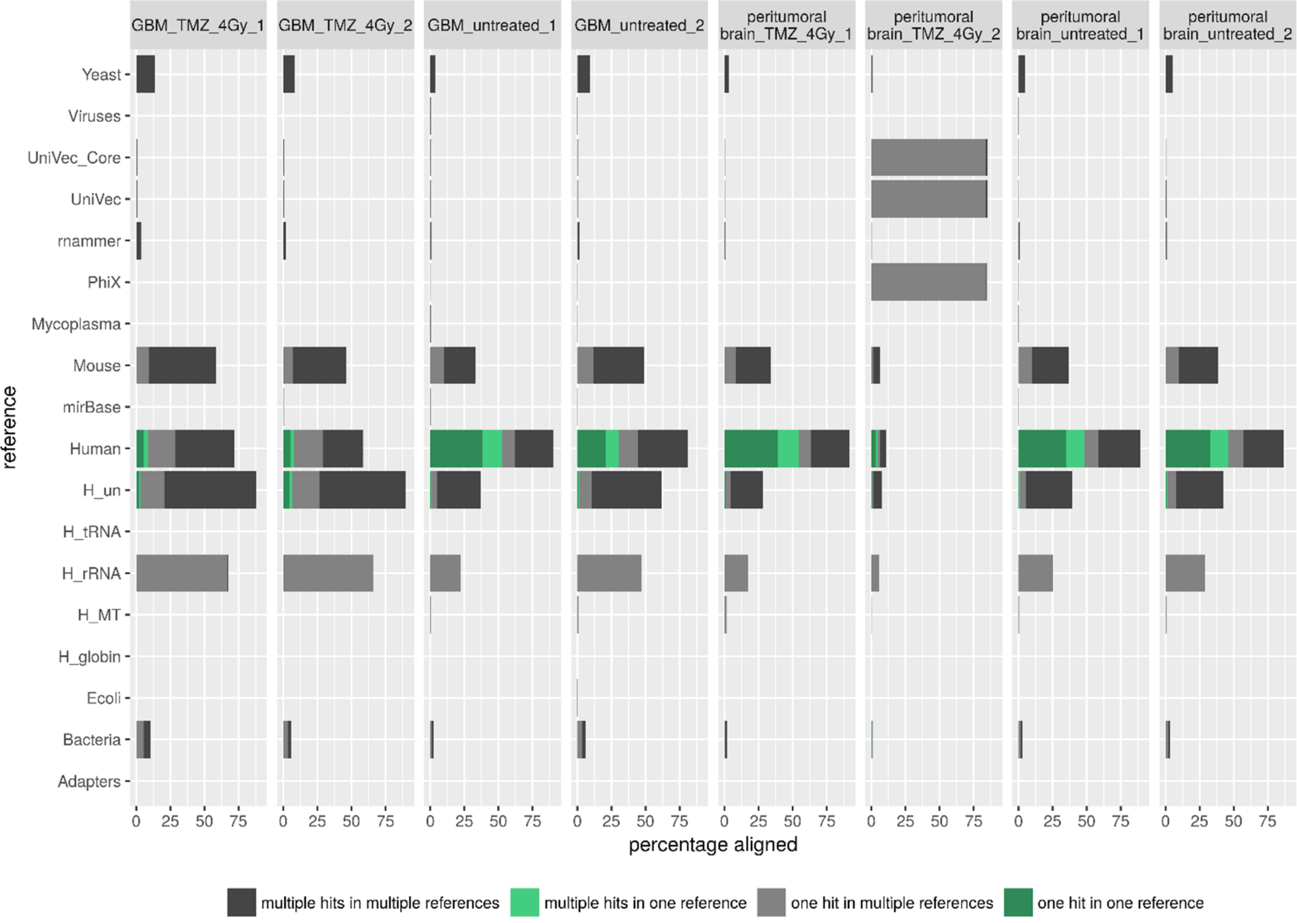

Suppl. Fig. S1: Quality Control of samples with respect to sequencing library composition. FastQ Screen v0.11.1a was used to assess sequencing library composition in order to detect possible contamination like bacteria and overrepresented fractions of RNA species like human rRNA. Values are given in percentage of reads aligning to selected sequence references. Reads are classified into four distinct types indicating reads uniquely mapping in one sequence reference (one hit in in one reference) , reads with multiple mappings in one sequence reference (multiple hits in one reference), reads uniquely mapping in distinct sequence references (one hit in multiple references), and reads with multiple mappings in distinct sequence databases (multiple hits in multiple references). The queried sequences are the following: Yeast (genome assembly SacCer3), Viruses (ftp://ftp.ncbi.nlm.nih.gov/genomes/refseq/viral/, March 2014), UniVec and UniVec Core (vector sequences from ftp://ftp.ncbi.nlm.nih.gov/pub/UniVec/ build 8.0, May 2015), rnammer (predicted rRNA sequences from http://www.cbs.dtu.dk/services/RNAmmer/, v1.2), phiX (gi|9626q372|ref|NC_001422.1| Enterobacteria phage phiX174 sensu lato, complete genome), Mycoplasma (ftp://ftp.ncbi.nih.gov/genomes/refseq/bacteria/Mycoplasma_* , Oct 2014), Mouse (genome assembly mm10), mirBase (miRNA sequences from mirBase v21), Human (human assembly GRCh37/hg19, reference chromosomes only), H_un (unplaced contigs and patches of GRCh37/hg19), H_tRNA (human transfer RNA sequences, http://gtrnadb.ucsc.edu/genomes/eukaryota/Hsapi19/hg19-tRNAs.fa), H_rRNA (human ribosomal RNA sequences, see Supplemental Table S1), H_MT (human mitochondrial reference sequence in GRCh37/hg19), H_globin (human hemoglobin mRNA sequences retrieved from NCBI RefSeq database, see Supplemental Table S2), Ecoli (Escherichia coli K12 DH10B), Bacteria (ftp://ftp.ncbi.nih.gov/genomes/refseq/bacteria/, Oct 2014), and adapters (adapter sequences from https://github.com/csf-ngs/fastqc/blob/master/Contaminants/contaminant_list.txt). Please note that the sample peritumoral brain_TMZ_4GY_2 is a dropout sample, since sequencing depth was about 50K reads and hence accumulates reads from the Illumina spike-in phiX.

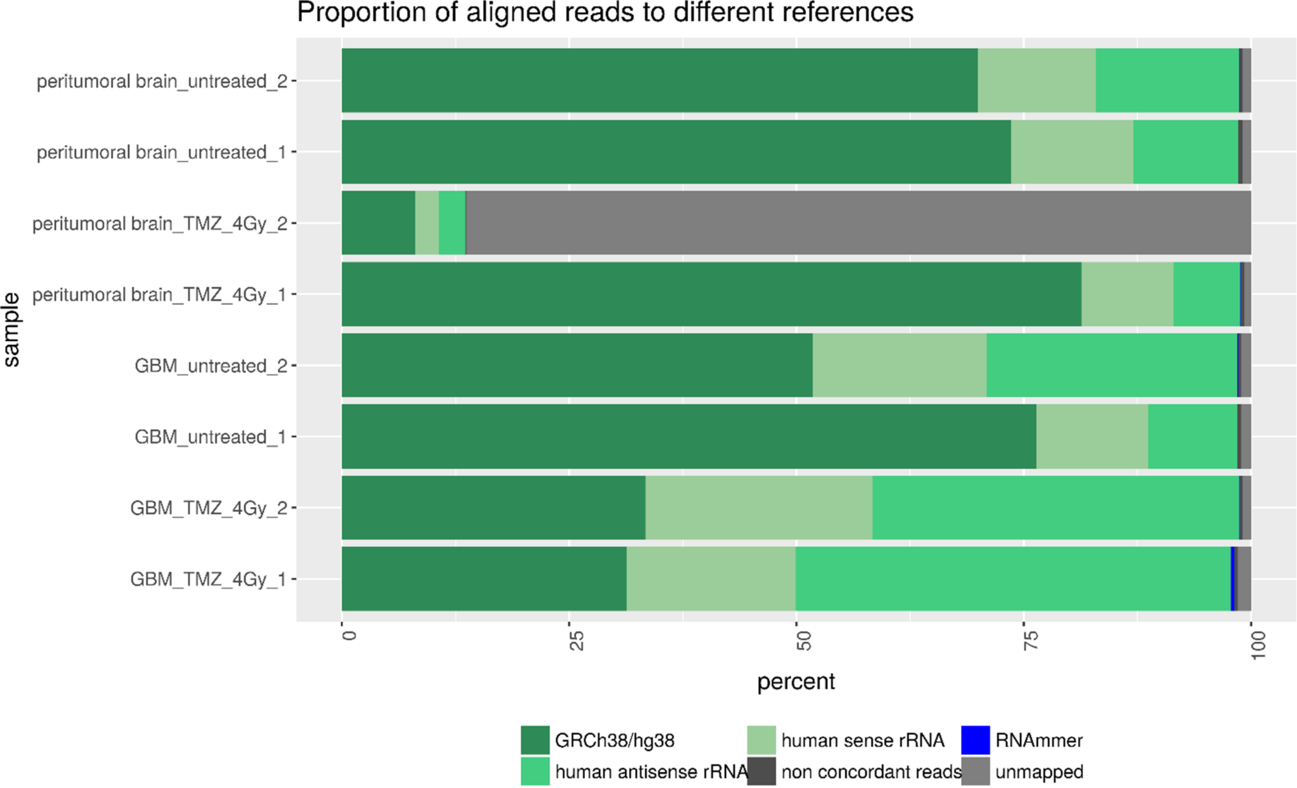

Suppl. Fig. 2: Quality Control of samples with respect to fraction of reads mapping to the human genome.

The fraction of reads mapping to the human genome was assessed iteratively by mapping reads against the RNAmmer database v1.2 (bowtie2 v2.2.7), human rRNA (bowtie2 v2.2.7), and the human genome assembly GRCh38/hg38 (segemehl v.0.2.0). Non-concordant reads represent reads which do not map linear to the human genome, but for example circular. Human rRNA reads are divided into sense, which resembles endogenous rRNA, and antisense rRNA which resembles rRNA antisense probes from the rRNA depletion step.

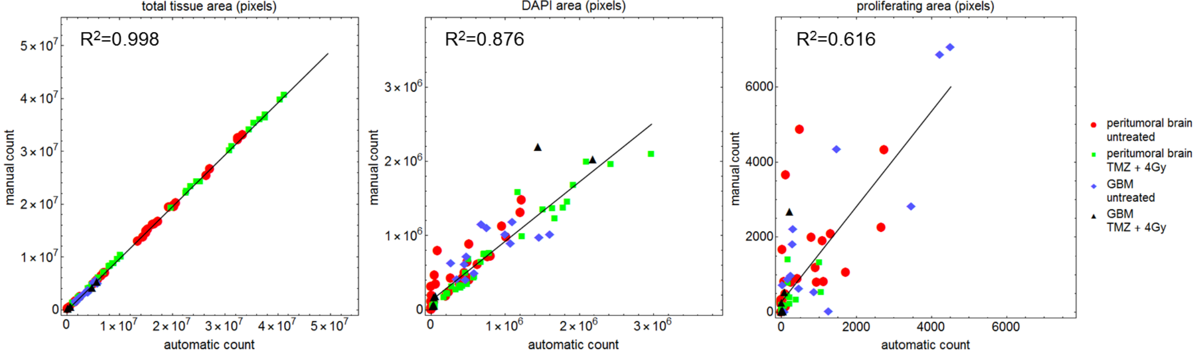

Suppl. Fig. 3: Correlation plots of manual versus automatic histological analysis.
The correlation coefficient R^2 of manual versus automatic counts were calculated for total tissue area (left plot), DAPI area (middle plot), and proliferating area (Ki67 positive, right plot). Red circles and green squares represent untreated and treated (TMZ+4Gy) peritumoral brain, whereas blue diamonds and black triangles represent untreated and treated (TMZ+4Gy) GBM tissue.
