# Supplementary material for: Deep sequencing and automated histochemistry of human tissue slice cultures improve their usability as preclinical model for cancer research
Source: Sci Rep. 2019 Dec 27;9:19961. doi: 10.1038/s41598-019-56509-5 (PMC6934722; doi:10.1038/s41598-019-56509-5)
Supplement: Supplementary file 2 — Supplementary Tables [file 41598_2019_56509_MOESM2_ESM.pdf]

Deep sequencing and automated histochemistry of human tissue slice cultures improve their usability as preclinical model for cancer research

Susann Haehnel[1*], Kristin Reiche[2], Dennis Loeffler[2], Andreas Horn[1], Conny Blumert[2], Sven-Holger Puppel[2], Nicole Kaiser[1], Felicitas Rapp[3], Michael Rade[2], Friedemann Horn[4,2], Juergen Meixensberger[5], Ingo Bechmann[1], Frank Gaunitz[5+] and Karsten Winter[1+]

[1]Institute of Anatomy, University of Leipzig, Faculty of Medicine, Germany

[2]Department of Diagnostics, Fraunhofer Institute of Cell Therapy and Immunology, Leipzig, Germany

[3]GSI Helmholtzzentrum für Schwerionenforschung GmbH, Darmstadt, Germany

[4]Institute of Clinical Immunology, University of Leipzig, Faculty of Medicine, Germany

[5]Department of Neurosurgery, University Hospital Leipzig, Germany

*corresponding author: su.haehnel@gmail.com

[+]contributed equally to this work

Tab. S1: rRNA sequences (NCBI RefSeq).

18S_gi	124517659	ref	NR_003286.1	Homo sapiens 18S ribosomal RNA (LOC100008588)
28S_gi	124517661	ref	NR_003287.1	Homo sapiens 28S ribosomal RNA (LOC100008589)
5S_gi	36084	emb	V00589.1	Human 5S ribosomal RNA
5.8S_gi	142372596	ref	NR_003285.2	Homo sapiens RNA, 5.8S ribosomal 1 (RN5-8S1), ribosomal RNA
12S_gi	251831106:648-1601			Homo sapiens mitochondrion, complete genome
16S_gi	251831106:1671-3229			Homo sapiens mitochondrion, complete genome

Tab. S2: NCBI RefSeq accession IDs for globins.

>gi	672228742	ref	NM_000558.4	Homo sapiens hemoglobin, alpha 1 (HBA1), mRNA
>gi	172072689	ref	NM_000517.4	Homo sapiens hemoglobin, alpha 2 (HBA2), mRNA
>gi	94538363	ref	NM_001003938.3	Homo sapiens hemoglobin, mu (HBM), mRNA
>gi	28302132	ref	NM_000184.2	Homo sapiens hemoglobin, gamma G (HBG2), mRNA
>gi	28302130	ref	NM_000559.2	Homo sapiens hemoglobin, gamm A (HBG2), mRNA
>gi	28302128	ref	NM_000518.4	Homo sapiens hemoglobin, beta (HBB), mRNA
>gi	6633805	ref	NM_005332.2	Homo sapiens hemoglobin, zeta (HBZ), mRNA
>gi	62865863	ref	NM_000519.3	Homo sapiens hemoglobin, delta (HBD), mRNA
>gi	28302129	ref	NM_005330.3	Homo sapiens hemoglobin, epsilon 1 (HBE1), mRNA
>gi	44955887	ref	NM_203378.1	Homo sapiens myoglobin (MB), transcript variant 3, mRNA
>gi	44955884	ref	NM_203377.1	Homo sapiens myoglobin (MB), transcript variant 2, mRNA
>gi	44955876	ref	NM_005368.2	Homo sapiens myoglobin (MB), transcript variant 1, mRNA

Tab. S3: Summary of the samples.

Sample	Zone	Treatment		Data
peritumoral brain	III	untreated	-	histology, RNA
		treated	TMZ + 4 Gy	histology
glioblastoma (GBM)	I	untreated	-	histology, RNA
		treated	TMZ + 4 Gy	histology, RNA
