# Supplementary material for: Deep sequencing and automated histochemistry of human tissue slice cultures improve their usability as preclinical model for cancer research
Source: Sci Rep. 2019 Dec 27;9:19961. doi: 10.1038/s41598-019-56509-5 (PMC6934722; doi:10.1038/s41598-019-56509-5)
Supplement: Supplementary file 3 — Supplementary Methods [file 41598_2019_56509_MOESM3_ESM.pdf]

Deep sequencing and automated histochemistry of human tissue slice cultures improve their usability as preclinical model for cancer research

Susann Haehnel[1*], Kristin Reiche[2], Dennis Loeffler[2], Andreas Horn[1], Conny Blumert[2], Sven-Holger Puppel[2], Nicole Kaiser[1], Felicitas Rapp[3], Michael Rade[2], Friedemann Horn[4,2], Juergen Meixensberger[5], Ingo Bechmann[1], Frank Gaunitz[5+] and Karsten Winter[1+]

[1]Institute of Anatomy, University of Leipzig, Faculty of Medicine, Germany

[2]Department of Diagnostics, Fraunhofer Institute of Cell Therapy and Immunology, Leipzig, Germany

[3]GSI Helmholtzzentrum für Schwerionenforschung GmbH, Darmstadt, Germany

[4]Institute of Clinical Immunology, University of Leipzig, Faculty of Medicine, Germany

[5]Department of Neurosurgery, University Hospital Leipzig, Germany

*corresponding author: su.haehnel@gmail.com

[+]contributed equally to this work

Supplementary Methods

Quality Control of Obtained Deep Sequencing Data

In order to assess the overall quality of the RNA sequencing for each tissue specimen a subsample of 1 million from the adapter-clipped first reads of the paired-end reads was randomly chosen by fastq-sample v0.0.14 using default parameters (https://github.com/dcjones/fastq-tools). The amount of clipped adapter sequences was evaluated with FastQC v0.11.5 (https://www.bioinformatics.babraham.ac.uk/projects/fastqc/), such that no adapter remnants should be detected and mean read quality for all bases should be above Q30 (Illumina Phred Quality Score, reflecting minimal base call accuracy of 99.9.%). All samples passed these two criteria. FastQ Screen v0.11.1a (https://www.bioinformatics.babraham.ac.uk/projects/fastq_screen/) was applied to detect possible contamination like bacteria and overrepresented fractions of RNA species like human rRNA. The queried sequences were comprised of bacterial genomes (ftp://ftp.ncbi.nih.gov/genomes/refseq/bacteria/, Oct 2014) including Escherichia coli (K_12 DH10B) and Mycoplasma species, virus genomes (ftp://ftp.ncbi.nlm.nih.gov/genomes/refseq/viral/, March 2014), the Illumina sequencing control (NCBI RefSeq accession gi|9626q372|ref|NC_001422.1| Enterobacteria phage phiX174 sensu lato, complete genome), the yeast genome (Saccharomyces cerevisiae S288C, assembly SacCer3), adapter sequences (https://github.com/csf-ngs/fastqc/blob/master/Contaminants/contaminant_list.txt), vector sequences (ftp://ftp.ncbi.nlm.nih.gov/pub/UniVec/ build 8.0, May 2015), predicted rRNA sequences (http://www.cbs.dtu.dk/services/RNAmmer/, v1.2), known human rRNA sequences (Supplemental Table S1), human tRNA sequences (http://gtrnadb.ucsc.edu/genomes/eukaryota/Hsapi19/hg19-tRNAs.fa), as well as

human globin sequences (Supplemental Table S2). For all samples, except one ("peritumoral brain_TMZ+4Gy_2"), only a negligible number of multi-mapping reads mapped to reference genomes other than human (see Supplemental Figure S1).

Since fastq-screen utilizes bowtie2 v2.2.7 for screening, which does not map split reads, we used in addition a stepwise alignment strategy to estimate the percentage of reads mapped to the human genome (version GRCh38/hg38). Firstly, reads were mapped against the RNAmmer v1.2 database reflecting predicted rRNAs in genomic sequences. Secondly, unaligned reads were mapped against the Escherichia coli genome. Thirdly, reads not aligned in the first two steps were mapped to human rRNA. NCBI RefSeq identifiers for the considered human rRNA genes are provided in Supplemental Table S1. Finally, remaining unaligned reads were mapped against the human genome (version GRCh38/hg38). Genomes without split features were aligned with bowtie v2.2.7 the other with segemehl v.0.2.0. Considering human rRNA and reads mapped to the human genome, alignment rates were above 97% which was deemed sufficient for analyses, although high rates of reads mapping to human sense or antisense rRNAs (17-66%) were observed. However, rRNA reads corresponding to endogenous rRNA resulted in a maintainable number of reads. The fraction of high reads mapping antisense to rRNA genes resembles rRNA antisense probes from the rRNA depletion step, and thus do not affect assessment of transcriptome variation (Supplemental Figure S2). In addition, principal component analysis of samples did not reveal any outliers, but shows a clear distinction between normal brain and tumor tissue (Figure 4D).
