# Supplementary material for: Deep sequencing and automated histochemistry of human tissue slice cultures improve their usability as preclinical model for cancer research
Source: Sci Rep. 2019 Dec 27;9:19961. doi: 10.1038/s41598-019-56509-5 (PMC6934722; doi:10.1038/s41598-019-56509-5)
Supplement: Supplementary file 4 — Supplementary Information [file 41598_2019_56509_MOESM4_ESM.pdf]

Deep sequencing and automated histochemistry of human tissue slice cultures improve their usability as preclinical model for cancer research

Susann Haehnel[1*], Kristin Reiche[2], Dennis Loeffler[2], Andreas Horn[1], Conny Blumert[2], Sven-Holger Puppel[2], Nicole Kaiser[1], Felicitas Rapp[3], Michael Rade[2], Friedemann Horn[4,2], Juergen Meixensberger[5], Ingo Bechmann[1], Frank Gaunitz[5+] and Karsten Winter[1+]

[1]Institute of Anatomy, University of Leipzig, Faculty of Medicine, Germany

[2]Department of Diagnostics, Fraunhofer Institute of Cell Therapy and Immunology, Leipzig, Germany

[3]GSI Helmholtzzentrum für Schwerionenforschung GmbH, Darmstadt, Germany

[4]Institute of Clinical Immunology, University of Leipzig, Faculty of Medicine, Germany

[5]Department of Neurosurgery, University Hospital Leipzig, Germany

*corresponding author: su.haehnel@gmail.com

[+]contributed equally to this work

```
# NeuroOrgano Hiseq
# 2016 11 04

destination_path: /mnt/fhgfs_ribdata/user_worktmp/sven-
holger.puppel/2016-Neuro-Organo/outHiSeq


constants:
    - &tophat2_genome_index_human_rRNA
/mnt/fhgfs_ribdata/references/human_rRNA/human_rRNA
#    - &tophat2_genome_index_human_mt
/mnt/fhgfs_ribdata/references/ hg19_mitochondrium/chrM
    - &tophat2_genome_index_rnammer
/mnt/fhgfs_ribdata/references/rRNA/rnammer-1.2
    - &segemehl_genome_index_hg38
/mnt/fhgfs_ribdata/references/hg38/hg38.idx
    - &genome_hg38  /mnt/fhgfs_ribdata/references/hg38/hg38.fa
    - &genome_faidx_hg38
/mnt/fhgfs_ribdata/references/hg38/hg38.fa.fai
    - &gencode_v25_hg38
/mnt/fhgfs_ribdata/annotations/hg38/gencode_v25/gencode.v25.an
notation.gtf
steps:


    fc1 (run_folder_source):
      path: /mnt/seqtransfer/RawData/Illumina/HiSeq/161104
_D00594_0053_AC6EAVANXX_KEEP/
      paired_end: True



    merge_tiles (merge_fastx_files):
         _depends: [fc1]
         _volatile: True


    AR (adapterremoval):
      _depends: [merge_tiles]
      cores: 10
      treatAs: paired
      adapter1: AGATCGGAAGAGCACACGTCT
      adapter2: AGATCGGAAGAGCGTCGTGTA
#      needs to be commented out there is no False flag
#      collapse: False
      trimns: True
      trimqualities: True
      minquality: 20
      threads: 10
      minlength: 20

    subsample (fastqsample):
      _depends: AR
      _connect:
        in/first_read:  AR/pair1.truncated
        in/second_read: AR/pair2.truncated
      n: 4000000
```

```
segemehl_hg38 (segemehl):
  _depends: AR
  _connect:
     in/second_read: AR/pair2.truncated
     in/first_read: AR/pair1.truncated
  index: *segemehl_genome_index_hg38
  genome : *genome_hg38
  _cluster:
    mem: 64G
    queue: pbatch
    exclusive: true
    time: 5-23:00:00

sort_by_name (sam_to_sorted_bam):
  _depends: segemehl_hg38
  sort-by-name: True
  genome-faidx: *genome_faidx_hg38
  temp-sort-dir: '/mnt/fhgfs_ribdata/user_worktmp/sven-
holger.puppel/2016-Neuro-Organo/sort-temp/sortdir1'

post_sawdust_hg38 (post_sawdust):
  _depends: sort_by_name
  library_type: 'fr-firststrand'
  seq_type: 'RNA'
  read_type: 'paired'
  _cluster:
    queue: pbatch
    time: 5-23:00:00


sort_by_chr_post_sawdust (sam_to_sorted_bam):
  _depends: post_sawdust_hg38
  genome-faidx: *genome_faidx_hg38
  temp-sort-dir: '/mnt/fhgfs_ribdata/user_worktmp/sven-
holger.puppel/2016-Neuro-Organo/sort-temp/sortdir2'




count_genes_fst_gencode25hg38 (htseq_count):
  _depends: post_sawdust_hg38
  stranded: 'reverse'
  mode: intersection-strict
  type: exon
  idattr: gene_id
  order: name
  feature-file: *gencode_v25_hg38
  _cluster:
    queue: pbatch
    time: 5-23:00:00


hg38_rseqc (rseqc):
   _depends: post_sawdust_hg38
```

```
       reference:
/mnt/fhgfs_ribdata/annotations/RSeQC/BED/Human_Homo_sapiens/hg
38_GENCODE_v23.bed

#    hg38_preseq_future_yield (preseq_future_yield):
#       _depends: sort_by_chr_post_sawdust
#       step:   1000000
#       extrap: 500000000
#       bootstraps: 1
#       pe: FALSE


    hg38_preseq_complexity_curve (preseq_complexity_curve):
       _depends: sort_by_chr_post_sawdust
       step: 100000
       pe: FALSE

    fastq_screen_fst (fastq_screen):
     _depends: subsample
     subset: 4000000
     config: /homes/olymp/sven-holger.puppel/Git/local/2016-
SmallRNA-Kits/pipeline/fastq_screen.conf
     cores: 32


    fastqc (fastqc):
       _depends: subsample
       _connect:
         in/second_read: 'empty'
#         in/first_read: AR/pair1.truncated
#                  in/second_read: AR/pair2.truncated




    subsample (fastqsample):
      _depends: AR
      _connect:
        in/first_read: AR/pair1.truncated
        in/second_read: 'empty'
      n: 1000000


    # AR2 (adapterremoval):
    #   _depends: [merge_tiles]
    #   cores: 10
    #   treatAs: paired
    #   adapter1: AGATCGGAAGAGCACACGTCT
    #   adapter2: AGATCGGAAGAGCGTCGTGTA
    #   collapse: True
    #   trimns: True
    #   trimqualities: True
    #   minquality: 20
    #   threads: 10
    #   minlength: 20
```

```
bowtie2_ecoli (bowtie2):
  _depends: subsample
  index: *tophat2_genome_index_rnammer


from_bowtie2_ecoli_fastq (sam_to_fastq):
  _depends: bowtie2_ecoli
  f: 4

bowtie2_human_rRNA (bowtie2):
  _depends: from_bowtie2_ecoli_fastq
  index: *tophat2_genome_index_human_rRNA
  _connect:
    in/second_read: 'empty'


bowtie2_human_rRNA_count (count_rRNA):
  _depends: bowtie2_human_rRNA


from_bowtie2_human_rRNA_fastq (sam_to_fastq):
  _depends: bowtie2_human_rRNA
  f: 4


segemehl_hg38_C (segemehl):
  _depends: from_bowtie2_human_rRNA_fastq
  _connect:
     in/second_read: 'empty'
  index: *segemehl_genome_index_hg38
  genome : *genome_hg38
  _cluster:
    mem: 64G
    queue: pbatch
    exclusive: true
    time: 5-23:00:00

sort_by_name_C (sam_to_sorted_bam):
  _depends: segemehl_hg38_C
  sort-by-name: True
  genome-faidx: *genome_faidx_hg38
  temp-sort-dir: '/mnt/fhgfs_ribdata/user_worktmp/sven-
holger.puppel/2016-Neuro-Organo/sort-temp/sortdir3'



post_sawdust_hg38_C (post_sawdust):
  _depends: sort_by_name_C
  library_type: 'fr-firststrand'
  seq_type: 'RNA'
  read_type: 'single'
```

```
tools:
#shell basics
        bam2mr:
            path: 'bam2mr'
            get_version: '--version'


        fastq-sample:
            path: 'fastq-sample'
            get_version: '--version'


        adapterremoval:
            path: 'AdapterRemoval'
            get_version: '--version'

        pwd:
            path: 'pwd'
            get_version: '--version'


        fastq_screen:
            path: '/homes/olymp/sven-
holger.puppel/Git/local/2016-SmallRNA-
Kits/pipeline/fastq_screen'
            get_version: '--version'

        pigz:
            path: 'pigz'
            get_version: '--version'

        echo:
            path: '/bin/echo'
            get_version: '--version'

        mkfifo:
            path: 'mkfifo'
            get_version: '--version'

        mkdir:
            path: 'mkdir'
            get_version: '--version'

        tar:
            path: 'tar'
            get_version: '--version'

        dd:
            path: 'dd'
            get_version: '--version'


        mv:
            path: 'mv'
            get_version: '--version'
```

```
        awk:
            path: 'awk'
            get_version: '--version'


        bowtie2:
            path: 'bowtie2'
            get_version: '--version'

        samtools:
            path: 'samtools'
            get_version: '--version'


        rm:
            path: 'rm'
            get_version: '--version'

        cut:
          path: 'cut'
          get_version: '--version'

        sort:
            path: 'sort'
            get_version: '--version'

        uniq:
            path: 'uniq'
            get_version: '--version'

        grep:
            path: 'grep'
            get_version: '--version'

        fix_qnames:
            path: '/homes/olymp/sven-
holger.puppel/Git/world/uap/tools/fix_qnames.py'
            get_version: '--help'
            exit_code: 0

        segemehl:
            path: 'segemehl.x'
            get_version: '--version'
            exit_code: 255

        post_sawdust:
          path: '/homes/olymp/sven-holger.puppel/Git/own/misc-
bio-stuff/scripts/post_sawdust.py'
          get_version: '-h'
          exit_code: 0


        bam_stat.py:
          path: bam_stat.py
          get_version: '--version'
          exit_code: 0
```

```
        infer_experiment.py:
          path: infer_experiment.py
          get_version: '--version'
          exit_code: 0

        read_distribution.py:
          path: read_distribution.py
          get_version: '--version'
          exit_code: 0


        preseq:
          path: preseq
          get_version: '--version'
          exit_code: 0

        htseq-count:
          path: htseq-count
          exit_code: 1


        cat:
          path: 'cat'
          get_version: '--version'

        fastq_screen:
          path: /homes/olymp/sven-
holger.puppel/Git/local/2016-SmallRNA-
Kits/pipeline/fastq_screen
          get_version: '--version'

        fastqc:
          path: fastqc
          get_version: '--version'
```
